# The Response of Growth and Transcriptome Profiles of Tea Grey Blight Disease Pathogen *Pestalotiopsis theae* to the Variation of Exogenous L-Theanine

**DOI:** 10.3390/ijms25063493

**Published:** 2024-03-20

**Authors:** Yuqian Zhang, Feiyan Wang, Lijie Wang, Lingyun Zhang, Richard V. Espley, Kui Lin-Wang, Fanrong Cao

**Affiliations:** 1 College of Horticulture, South China Agricultural University, Guangzhou 510642, China; yuqianzhang@scau.edu.cn (Y.Z.); wanglijie20210726@163.com (L.W.); lingyunzh@163.com (L.Z.); 2 Key Laboratory of Biology and Genetic Improvement of Horticultural Crops (South China), Ministry of Agriculture and Rural Affairs, College of Horticulture, South China Agricultural University, Guangzhou 510642, China; 3 Institute of Biological and Medical Engineering, Guangdong Academy of Sciences, Guangzhou 510316, China; wfei08@163.com; 4 New Zealand Institute for Plant & Food Research Limited, Auckland 1142, New Zealand; richard.espley@plantandfood.co.nz (R.V.E.); kui.lin-wang@plantandfood.co.nz (K.L.-W.)

**Keywords:** L-theanine, *Pestalotiopsis theae*, tea grey blight disease, growth inhibition, transcriptome

## Abstract

Tea grey blight disease is one of the most destructive diseases that infects tea and is caused by the pathogen *Pestalotiopsis theae* (Sawada) Steyaert. L-theanine is a unique non-protein amino acid of the tea plant. Different concentrations of L-theanine exhibit significant inhibitory effects on the growth and sporulation ability of the pathogen causing tea grey blight disease. To understand the effect mechanism of L-theanine on *P. theae*, transcriptome profiling was performed on the pathogenic mycelium treated with three different concentrations of L-theanine: no L-theanine treatment (TH0), 20 mg/mL theanine treatment (TH2), and 40 mg/mL theanine treatment (TH4). The colony growths were significantly lower in the treatment with L-theanine than those without L-theanine. The strain cultured with a high concentration of L-theanine produced no spores or only a few spores. In total, 2344, 3263, and 1158 differentially expressed genes (DEGs) were detected by RNA-sequencing in the three comparisons, Th2 vs. Th0, Th4 vs. Th0, and Th4 vs. Th2, respectively. All DEGs were categorized into 24 distinct clusters. According to GO analysis, low concentrations of L-theanine primarily affected molecular functions, while high concentrations of L-theanine predominantly affected biological processes including external encapsulating structure organization, cell wall organization or biogenesis, and cellular amino acid metabolic process. Based on KEGG, the DEGs of Th2 vs. Th0 were primarily involved in pentose and glucuronate interconversions, histidine metabolism, and tryptophan metabolism. The DEGs of Th4 vs. Th0 were mainly involved in starch and sucrose metabolism, amino sugar, and nucleotide sugar metabolism. This study indicated that L-theanine has a significant impact on the growth and sporulation of the pathogen of tea grey blight disease and mainly affects amino acid metabolism, carbohydrate metabolism, and cellular structure-related biosynthesis processes of pathogenic fungi. This work provides insights into the direct control effect of L-theanine on pathogenic growth and also reveals the molecular mechanisms of inhibition of L-theanine to *P. theae*.

## 1. Introduction

Tea (*Camellia sinensis*) is a major crop primarily used in the food and beverage industry. However, tea plants are threatened by many diseases [1]. Diseases cause annual tea yield losses along with quality reduction, with blister blight disease causing 43% losses of yield [2]. Tea grey blight disease is one of the most destructive diseases and is caused by the pathogen *Pestalotiopsis theae* (Sawada) Steyaert [3]. Tea grey blight disease can seriously harm tea leaves and new shoots of the tea plant and significantly reduce the tea yield [3]. There is currently a lack of environmentally safe and effective prevention and control strategies for this disease. Tea is rich in metabolites of L-theanine, polyphenols, alkaloids, tea saponin, and tea polysaccharides [4]. These metabolites not only have functions in nutrients and healthcare for humans but also play an important role in plant health and development processes [5,6]. Tea polyphenols and caffeine have various biological activities, such as antioxidation, and inhibitory effects on fungi [7]. L-theanine can pass through the blood–brain barrier quickly [8] and exhibit a neuroprotective effect against cerebral ischemia-induced nerve cell apoptosis [9]. Tea polyphenols have antibacterial properties [10], and lignin, an important physical antimicrobial substance, produces lignification to prevent the spread of pathogens [11]. Thus, these metabolites are beneficial to the growth, disease resistance, and stress resistance of tea plants.

L-theanine (N-ethyl-γ-glutamine) is one of the most abundant free nitrogenous compounds and a unique non-protein amino acid. It is the most abundant free amino acid (more than half of the total free amino acids) in the tea plant [12]. When the tea plant goes through winter, the tea plant root system can synthesize a large amount of L-theanine along with the process of absorbing and storing nitrogen. With the onset of spring, L-theanine was transported to leaf tissues with different development levels, such as new shoots [13,14]. It makes an important contribution to the quality of tea, such as enhancing the umami taste of tea soup and reducing its bitter taste [15]. L-theanine has multiple functions, not only for humans (such as exhibiting a neuroprotective effect [9,16] and facilitating learning abilities [17,18]), but also for the tea plant (like inhibiting denitrification and nitrification and reducing the relative abundance of some microbial genera or phyla such as *Mycobacterium*, *Alicyclobacillus*, *Dyella* and Firmicutes phylum [19,20,21,22,23,24]). In human and animal research, L-theanine can alleviate or eliminate inflammation in humans or animals, which means it has a significant inhibitory effect on microorganisms [25,26,27]. In plant diseases, 2.5 g/L. L-theanine can significantly reduce the growth rate of *Colletotrichum coccodes* [28].

The nitrate reductase-related genes (*narH*, *napA*, and *napB*) contribute to denitrification and complete nitrification, in which the content of nitrogen could be affected in the rhizosphere soil by these genes. In the study of the interaction between plants and L-theanine, one published study showed that L-theanine can affect the abundance of these functional genes (*narH*, *napA*, and *napB*) and some other key genes involved in the cycle of carbon, nitrogen, and phosphorus [19]. Therefore, L-theanine appears to function as both a storage and nitrogen carrier, producing other organic compounds such as glutamate (Glu) and glutamine (Gln) based on soil nitrogen levels. In turn, a study demonstrates that ammonium fertilizer application was more efficient in increasing the L-theanine content in the xylem than nitrate [20]. L-theanine biosynthesis and accumulation can be affected by inorganic nitrogen in soils. The tea plant root system shows a preference for ammonium (NH_4_^+^) over nitrate (NO_3_^−^), and NH_4_^+^ is more easily assimilated into L-theanine than NO_3_^−^ [21,22,23]. In other plants, nitrogen metabolism differs from tea plants due to the unique L-theanine metabolism pathway in the tea plant [20,21]. L-theanine can significantly increase or decrease rhizosphere microbial diversity. For example, different concentrations of L-theanine have different effects on increasing the relative abundance of microbial phyla, and it can also decrease the relative abundance of Firmicutes and some gena such as *Burkholderia* [19]. Glutamine and L-theanine have similar chemical structures and also show the same inhibitory effect of Firmicutes [24]. However, the research on the effects of L-theanine mainly focuses on the health functions of humans and its effects on plant nitrogen metabolism and microbial diversity. There are almost no reports on the inhibitory effect of L-theanine on plant pathogens or the improvement of plant disease resistance as an exogenous substance in vitro culture or plants.

In the present study, we found that L-theanine not only inhibits the growth of the tea grey blight disease pathogen but also seriously affects the production of pathogenic spores. The effects of different concentrations of L-theanine on pathogens are inconsistent, and the higher the concentration of L-theanine, the more significant its impact on the pathogen. We subsequently investigated how the exogenous addition of L-theanine with different concentrations alters the gene expression pattern of the pathogen. An RNA-seq analysis was conducted on pathogenic mycelium to dissect the transcriptomic profiles in response to treatment with different concentrations of L-theanine. GO (Gene Ontology), KEGG (Kyoto Encyclopedia of Genes and Genomes) pathway analysis, and KOG (EuKaryotic Orthologous Groups) of RNA-seq data were employed to decipher the network of metabolic and signaling pathways involved in the regulation of growth of pathogenic hyphae and production of spores in response to L-theanine. The analysis specifically focused on the expression profiles of genes related to hyphal cell structure and spore formation. To the best of our knowledge, this is the first RNA-seq-based study to assess the effect of L-theanine on the transcriptomic profiles, and the sequencing data contributed to understanding the molecular effects exerted by L-theanine on tea grey blight disease pathogenic hyphae and spores.

## 2. Results

### 2.1. Phenotypic Analysis Revealing the Inhibitory Effects of L-Theanine

**Figure 1:**
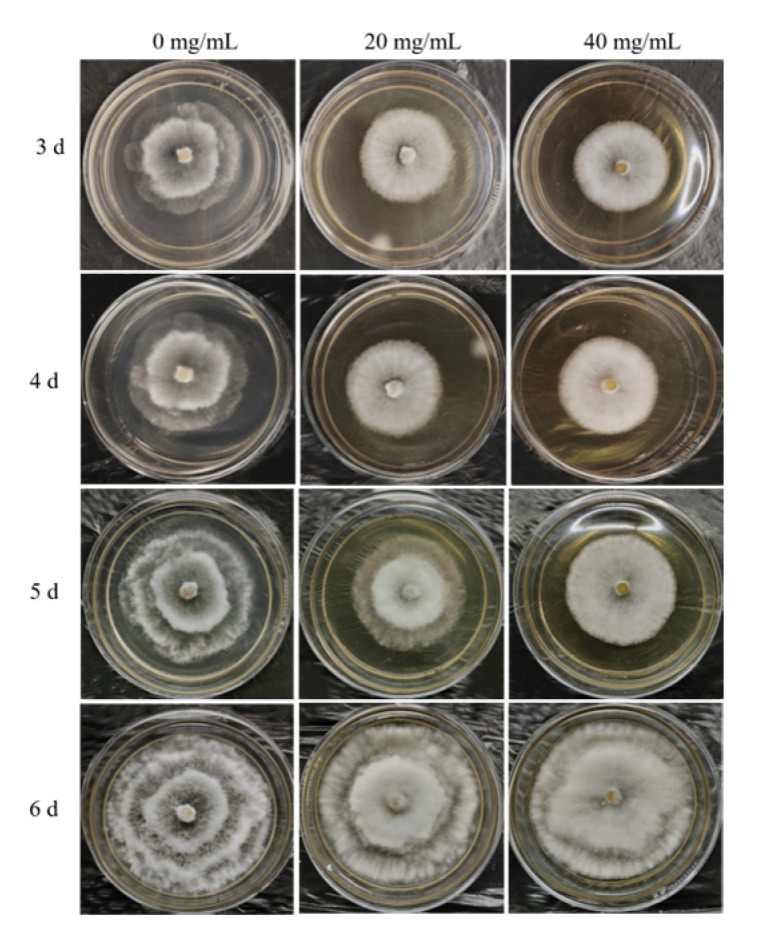
Growth phenotypes of the pathogenic fungus *Pestalotiopsis theae* in a medium containing different concentrations of L-theanine (0, 20, and 40 mg/L). The photos were taken at 3, 4, 5, and 6 days after the pathogenic fungal cakes were cultured on PDA plates.

**Figure 2:**
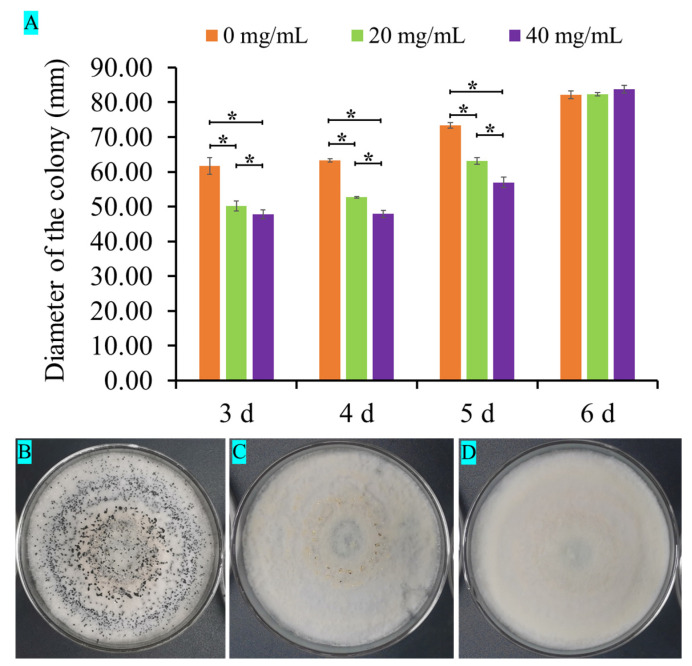
Statistical analysis of colonial diameters and sporulation pattern of *Pestalotiopsis theae* in a medium containing different concentrations of L-theanine. (**A**) Statistical analysis of colonial diameters. (**B**–**D**) Sporulation pattern of *Pestalotiopsis theae* at 12 days. Values indicate the *p*-value (significant at *p*-value < 0.05) of the results of pairwise comparison using ANOVA (*: adjust *p*-value < 0.05).

**Figure 3:**
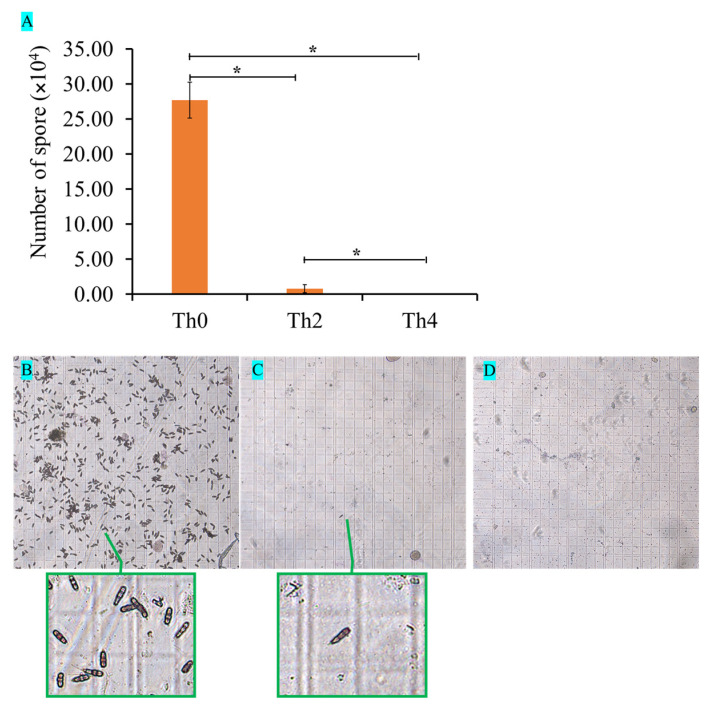
Statistical analysis of *Pestalotiopsis theae* spores in a medium containing different concentrations of L-theanine. (**A**) Quantity statistics of spores. (**B**–**D**) Microscopic observation of spores at 12 days in the blood counting chamber. Values indicate the *p*-value (significant at *p*-value < 0.05) of the results of pairwise comparison using ANOVA (*: adjust *p*-value < 0.05).

When the fresh pathogenic fungal cakes were cultured on PDA plates with different concentrations of L-theanine for 3 days, the colonial diameters were measured by Vernier caliper and photographed from day 3 to day 6. According to the statistical analysis of colonial diameters, there was a significant difference in the colonial diameters from day 3 to day 5 among the three different concentrations of L-theanine treatment. The colony growths were significantly lower in the medium containing L-theanine than in the medium without L-theanine within 5 days (Figure 1 and Figure 2A). After the 6th day, the mycelium covered the entire culture dish. Although the colony diameter was measured, it was not possible to truly compare the differences among different treatments. Another interesting phenomenon was that the strain cultured in the medium containing high L-theanine concentration produced no spores and produced a few spores in the low-concentration medium when the fungus was continuously cultured for 12 days (Figure 2C,D and Figure 3A,C,D). However, the strain cultured in a medium without L-theanine produced a large number of spores (Figure 2B and Figure 3A,B).

### 2.2. Differential Expression of Genes in the Three Comparisons

**Figure 4:**
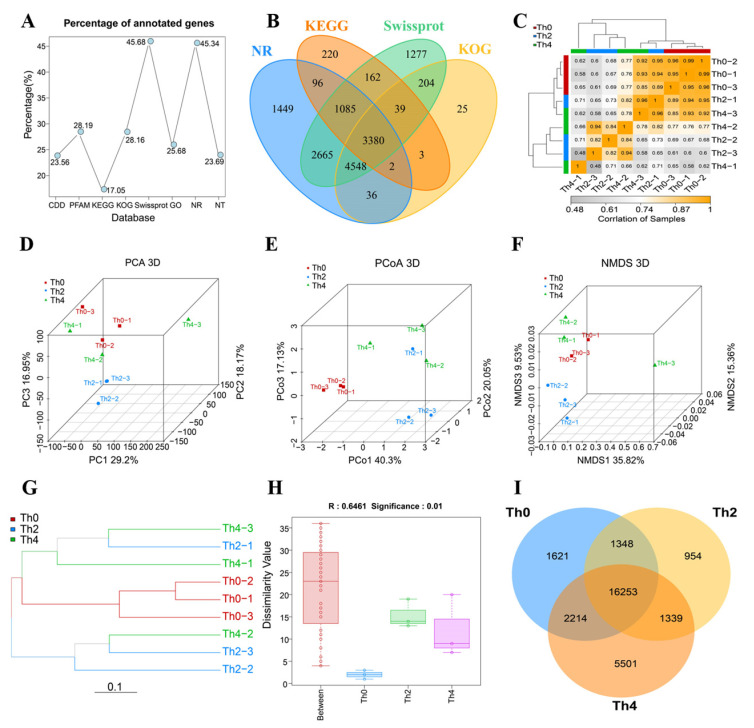
Gene annotation and overall differences at the transcript level among the samples. (**A**,**B**) Gene annotation in eight functional annotation databases. (**C**) Correlation coefficient of biological replicates. (**D**) Principal component analysis (PCA). (**E**) Principal coordinates analysis (PCoA). (**F**) Nonmetric multidimensional scaling (NMDS). (**G**) Hierarchical cluster analysis. (**H**) Anosim analysis. (**I**) Venn diagrams of unigenes in the three samples.

**Figure 5:**
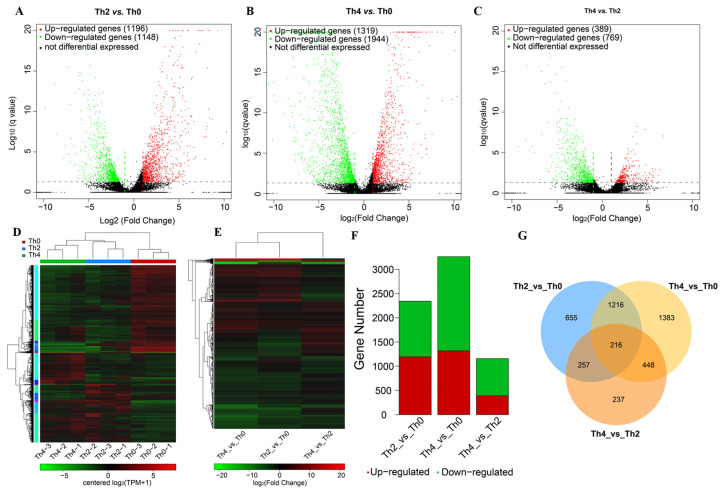
Total down- and up-regulated genes in the three comparisons. (**A**–**C**) Volcano plots of the differentially expressed genes in the three comparisons (Th2 vs. Th0, Th4 vs. Th0, and Th4 vs. Th2), up/down-regulated expression levels of genes were presented by red/green dots, and black dots indicate no difference. (**D**) Expression heatmap of all identified and detected unigenes from the transcriptome. (**E**) Expression heatmap of DEGs in the three comparisons. (**F**) Status of down/upregulated genes. (**G**) Venn diagrams of DEGs in the three comparisons.

To identify differentially expressed genes of pathogenic fungi treated with different concentrations of L-theanine, reference-free transcriptome analysis was carried out. RNA-Seq produced 48.99–58.86 (Q30 Bases Ratio = 91.70–92.96%), 56.31–60.16 (Q30 Bases Ratio = 91.79–92.64%), 54.84–58.96 (Q30 Bases Ratio = 92.4–94.20%) million raw reads from Th0 to Th4 cDNA libraries. After stringent quality checks and data clean-up, the clean reads of Th0, Th2, and Th4 groups were 44.61–54.28 (94.87–95.35%), 52.37–55.27 (94.72–95.15%), and 52.01–54.50 (94.72–95.15%), respectively (Table S1). There were 29,247 unigenes identified in the transcriptome data (Table S2). All unigenes were carried out functionally annotated in CDD, PFAM, KEGG, KOG, Swissport, GO, NR, and NT databases; 45.68 and 45.34 percent of unigenes were annotated in Swissport and NR databases, respectively (Figure 3B and Figure 4A). Swissport and NR were databases with the largest proportional number of unigenes (Figure 4B). The clean reads were mapped to the reference sequence (Unigenes database) by using Bowtie2. In total, 43.71–53.10, 40.96–61.07, and 35.23–51.36 million reads were mapped to the unigenes database, with match ratios in the ranges of 98.97–99.07%, 98.75–98.85%, and 98.63–98.94% in Th0, Th2, and Th4 samples, respectively (Table S1). We identified and detected 21,436, 19,894, and 25,307 genes in Th0, Th2, and Th4 samples, respectively (Table S2, Figure 4I and Figure 5D). A high correlation coefficient (R^2^ > 0.99) of gene expression between biological replicates indicated the effectiveness of the data (Figure 4C). Hierarchical cluster analysis revealed obvious clear differences in genes among samples treated with different concentrations of L-theanine (Figure 4G) and was consistent with PCA (PC1 = 29.2%), PCoA (PCo1 = 40.3%), and NMDS (NMDS1 = 35.82%) (Figure 4D–F). The result of the Anosim analysis shows a significant difference between the three fungus sample groups (R = 0.6461, *p*-value < 0.01, Figure 4H).

With the filter criteria of |log_2_FoldChange| ≥ 2 and false discovery rate (*q*-value) < 0.05, in total, 4412 differentially expressed genes (DEGs) of pathogenic fungus were identified through the three treatments of L-theanine (Tables S3–S5), and there were 2344, 3263, and 1158 differentially expressed genes (DEGs) detected in the three comparisons (Th2 vs. Th0, Th4 vs. Th0, and Th4 vs. Th2, respectively), of which 1196, 1319, and 389 DEGs were upregulated and 1148, 1944, and 769 DEGs were downregulated in the three comparison groups (Tables S3–S5, Figure 5A–C,E,F). Venn diagram analysis shows that 216 DEGs were common to the three comparisons (Figure 5G). When the concentration of L-theanine was increased, there were many differentially expressed genes (1216 DEGs) that responded together (Figure 5G). When pathogens were treated with different concentrations of L-theanine, different alignment groups also had their own differentially expressed genes (Figure 5G).

**Figure 6:**
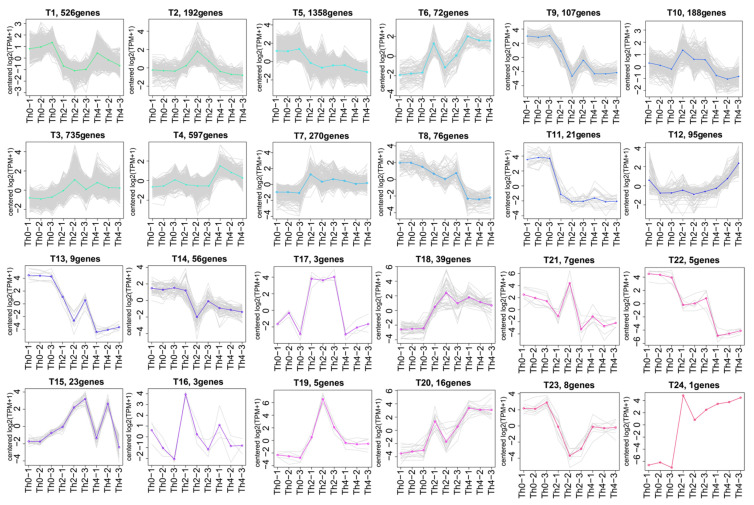
K-means clustering analysis of all DEGs in the three comparisons.

**Figure 7:**
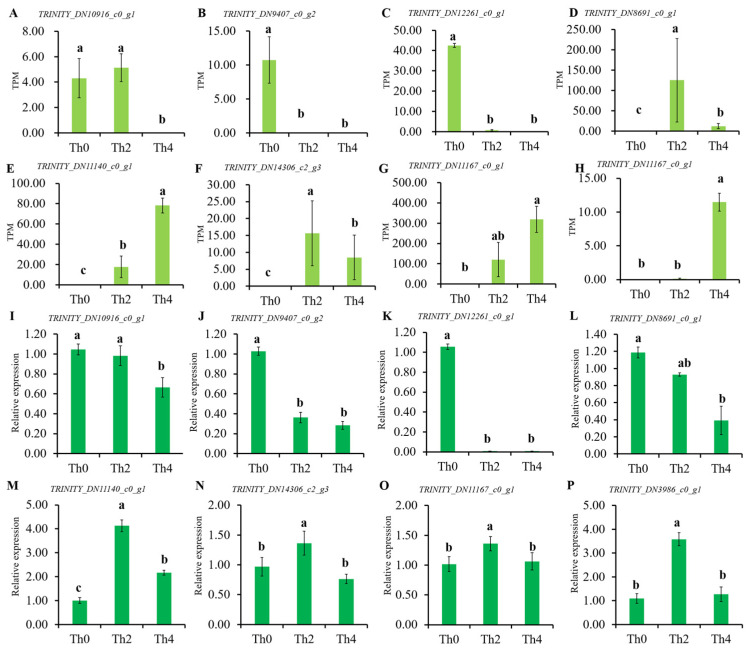
Differentially expressed genes (DEGs) in Th2 vs. Th0, Th4 vs. Th0, and Th4 vs. Th2 comparison groups. (**A**–**H**) Differentially expressed genes by transcriptome. (**I**–**P**) qRT-PCR showing the expression levels of eight DEGs in the three comparisons.

Further, k-means clustering analysis of 4412 DEGs exhibited 24 distinct clusters (T1–T24) corresponding to three treatments with different concentrations of L-theanine to *P. theae*: Th0 (T1, T5, T8, T9, T11, T13, T14, T22, T23), Th2 (T2, T3, T7, T10, T15–T19, T21, T24), and Th4 (T4, T6, T12, T20), suggesting that the high-expression patterns of identified genes were diverse throughout three treatments with different concentrations of L-theanine (Figure 6; Table S6). While inspecting the potential roles of DEGs, the high-expression genes in Th0 (shown by T1, T5, T8, T9, T11, T13, T14, T22, T23) were mainly involved in signal transduction mechanisms, lipid transport and metabolism, secondary metabolites biosynthesis, transport and catabolism, amino acid transport and metabolism, cell wall/membrane/envelope biogenesis, and energy production and conversion; high-expression genes in Th2 (shown by T2, T3, T7, T10, T15–T19, T21, T24) were mainly involved in cell wall/membrane/envelope biogenesis, carbohydrate transport and metabolism, secondary metabolites biosynthesis, transport and catabolism, energy production and conversion, cytoskeleton, lipid transport and metabolism, signal transduction mechanisms, and defense mechanisms; high-expression genes in Th4 (shown by T6 T4, T12, T20) were mainly involved in amino acid transport and metabolism, inorganic ion transport and metabolism, secondary metabolites biosynthesis, transport and catabolism, posttranslational modification, protein turnover, chaperones, translation, ribosomal structure and biogenesis, cell cycle control, cell division, chromosome partitioning, carbohydrate transport and metabolism, nucleotide transport and metabolism, and coenzyme transport and metabolism (Tables S3–S5). The top eight DEGs (TRINITY_DN11140_c0_g, 1TRINITY_DN11167_c0_g1, TRINITY_DN14306_c2_g3, TRINITY_DN3986_c0_g1, TRINITY_DN8691_c0_g1, TRINITY_DN9407_c0_g2, TRINITY_DN12261_c0_g1, and TRINITY_DN10916_c0_g1) were screened to validate the transcriptomic results. The qRT-PCR results showed that TRINITY_DN11140_c0_g, 1TRINITY_DN11167_c0_g1, TRINITY_DN14306_c2_g3, and TRINITY_DN3986_c0_g1 were lower in Th0 than that in Th2, and Th4; TRINITY_DN9407_c0_g2, TRINITY_DN12261_c0_g1, and TRINITY_DN10916_c0_g1 were suppressed by L-theanine (Figure 7). These results were generally consistent with transcriptome data.

### 2.3. Gene Ontology Analysis of Differentially Expressed Genes

The gene ontology (GO) analysis was conducted to investigate the roles of genes related to the response of pathogenic fungi to L-theanine in the biological process (BP), cell component (CC), and molecular function (MF) terms. For the changes in gene expression levels during the L-theanine stress response process, the GO analyses of 2344 DEGs in Th2 vs. Th0 comparison were mainly enriched in catalytic activity (*p*-value = 9.80 × 10^−15^), mitochondrion (*p*-value = 4.10 × 10^−9^), 2-aminoadipate transaminase activity (*p*-value = 1.00 × 10^−6^), oxidoreductase activity (*p*-value = 5.90 × 10^−6^), and cellular amino acid metabolic process (*p*-value = 7.70 × 10^−7^); 3263 DEGs in Th4 vs. Th0 comparison were mainly enriched in catalytic activity (*p*-value = 2.30 × 10^−13^), fungal-type vacuole (*p*-value = 2.40 × 10^−11^), storage vacuole (*p*-value = 2.70 × 10^−11^), fungal-type cell wall organization or biogenesis (*p*-value = 3.90 × 10^−11^), and external encapsulating structure organization (*p*-value = 3.00 × 10^−10^); 1158 DEGs in Th4 vs. Th2 comparison were mainly enriched in cellular component (*p*-value < 0.05), such as endoplasmic reticulum membrane (*p*-value = 6.80 × 10^−4^) and fungal-type vacuole (*p*-value = 1.10 × 10^−3^) (Table S7, Figures S1, S4 and S7). At low concentrations of L-theanine (Th2), some other genes that involved in cellular components, and biological processes were significantly affected by L-theanine, including TRINITY_DN14134_c0_g1, TRINITY_DN9580 c0g1, TRINITY_DN11891_c0_g1, TRINITY_DN1554_c0_g1, and TRINITY_DN2717_c0_g1 (Figures S2 and S3). However, most genes that were affected by L-theanine were involved in molecular function, including catalytic activity and oxidoreductase activity such as TRINITY_DN4336_c0_g1, TRINITY_DN7940_c0_g1, TRINITY_DN14240_c0_g1, TRINITY_ DN23344_c0_g1, and TRINITY_DN9305_c0_g1 (Figure S3). At high concentrations of L-theanine (Th4), most genes that were affected by L-theanine were mainly involved in biological processes, including external encapsulating structure organization (*p*-value = 3.00 × 10^−10^), cell wall organization or biogenesis (*p*-value = 5.40 × 10^−10^), cellular amino acid metabolic process (*p*-value = 9.00 × 10^−10^) and molecular functions such as catalytic activity (*p*-value = 2.30 × 10^−13^) (Figures S5 and S6). The significantly differentially expressed genes included TRINITY_DN9614_c0_g1, TRINITY_DN10867_c0_g1, TRINITY_ DN13678_c0_g1, and TRINITY_DN8588_c0_g1. The differences in transcription levels between the two concentrations of L-theanine mainly were the cellular components including endoplasmic reticulum membrane (*p*-value = 6.80 × 10^−4^), and fungal-type vacuole (*p*-value = 1.10 × 10^−3^). The significantly differentially expressed genes include TRINITY_DN6207_c0_g1, TRINITY_DN12047_c0_g1, and TRINITY_DN14296_c0_g1 (Figures S7–S9).

### 2.4. KEGG Analysis of Differentially Expressed Genes

KEGG analysis (*p*-value < 0.05) revealed that all of these DEGs in all comparisons were mainly enriched in 27 metabolic processes. The top ten significantly enriched metabolic pathways include pentose and glucuronate interconversions, histidine metabolism, tryptophan metabolism, phenylalanine metabolism, isoquinoline alkaloid biosynthesis, starch and sucrose metabolism, tropane, piperidine and pyridine alkaloid biosynthesis, tyrosine metabolism, beta-alanine metabolism, and arginine and proline metabolism in Th2 vs. Th0; and starch and sucrose metabolism, aminobenzoate degradation, tryptophan metabolism, phenylalanine metabolism, tyrosine metabolism, isoquinoline alkaloid biosynthesis, amino sugar and nucleotide sugar metabolism, pentose and glucuronate interconversions, pentose phosphate pathway, and histidine metabolism in Th4 vs. Th0 (Table S8 and Figures S10 and S11). The different gene expressions of pathogen fungus treated with two concentrations of L-theanine were shown in various types of N-glycan biosynthesis and peroxisome (Table S8 and Figure S12). Different concentrations of L-theanine mainly affected the metabolism of amino acids, alkaloids, pentose, starch, and sucrose. Among these DEGs detected in Th2 vs. Th0 and Th4 vs. Th0, 23 DEGs were involved in alkaloid biosynthesis, 58 DEGs were involved in starch and sucrose metabolism, and 65 DEGs were involved in amino acid metabolism (Figures S10–S12). When the concentrations of L-theanine reached the Th4 level, another 49 DEGs that were involved in aminobenzoate degradation, amino sugar, and nucleotide sugar metabolism, and pentose phosphate pathways were affected by L-theanine, including glyco_hydro_61 gene (TRINITY_DN14362_c0_g1 (log2FC(Th2 vs. Th0) = −6.09; log2FC (Th4 vs. Th0) = −6.92)), tyosinase_c gene (TRINITY_DN8043_c0_g1 (log2FC (Th2 vs. Th0) = −4.15; log2FC (Th4 vs. Th0) = −8.11)), and copper amine oxidase gene (TRINITY_DN13980_c2_g1 (log2FC (Th2 vs. Th0) = −4.09; log2FC (Th4 vs. Th0) = −4.26)).

### 2.5. KOG Analysis of Differentially Expressed Genes

KOG analysis revealed that all of these DEGs in all comparisons were mainly enriched in eight metabolic processes (*p*-value < 0.05) including general function prediction only, secondary metabolites biosynthesis, transport and catabolism, cell wall/membrane/envelope biogenesis, lipid transport and metabolism, amino acid transport and metabolism, inorganic ion transport and metabolism, carbohydrate transport and metabolism, and energy production and conversion (Table S9 and Figures S13–S15). There were 1071 genes involved in these metabolic and biosynthetic processes such as FAD-linked oxidoreductase gene (TRINITY_DN6909_c0_g2 (log2FC (Th2 vs. Th0) = −13.95; log2FC (Th4 vs. Th0) = −13.95)), amino acid transporter-related gene (TRINITY_DN6763_c0_g2 (log2FC (Th2 vs. Th0) = −13.52; log2FC (Th4 vs. Th0) = −13.52)), and cysteine synthase (TRINITY_DN11167_c0_g1 (log2FC (Th2 vs. Th0) = 20.20; log2FC (Th4 vs. Th0) = 21.61)).

## 3. Discussion

A multicellular fungus is composed of mycelia and spores. Mycelia and spores are the main basis for fungal classification and identification [29]. Mycelia are the main organs that can absorb nutrients from the growing environment while spores are the main reproductive organs. The metabolic pathways of fungi involved in the formation of mycelia and spores mainly include organic acid metabolism, protein metabolism, lipid metabolism, glucose metabolism, and so on [30]. Among them, sugar metabolism is the main source of energy for fungi, in which other sugars are converted into energy through glycolysis reactions, contributing to both the development and reproduction of fungi [31]. Fungal growth can be influenced by various factors, such as light, temperature, and nutrients [32]. Also, fungal growth can be inhibited by other compounds, whether from plants or synthesized, especially for plant pathogens. Plant pathogens can be inhibited by various metabolites of plants, such as flavonoids, alkaloids, terpenoids, and amino acids [33,34]. In the present research, the growth rate of pathogenic mycelia was significantly inhibited by L-theanine within 5 days. Due to the limitations of the culture dish, the mycelium rapidly grew throughout the entire dish on the 6th day, which made it impossible to truly compare differences in colony diameter among different treatments. Additionally, when the pathogenic fungus was treated with L-theanine, the formation of pathogenic spores was significantly inhibited, and even 40 mg/mL L-theanine can completely inhibit formation. Based on transcriptome data, 40 mg/L of L-theanine further affects amino acid metabolism, starch and sucrose metabolism, and amino sugar and nucleotide sugar metabolism. These metabolisms were involved in some downregulated genes such as cysteine synthase gene (TRINITY_DN4280_c0_g1), cysteine dioxygenase gene (TRINITY_DN10540_c0_g1), and endo-chitinase gene (TRINITY_DN9963_c0_g1, TRINITY_DN10305_c0_g1), and they are related to fungal sporulation ability [35,36,37,38]. Thus, it may be due to these key genes involved in these metabolic pathways that higher concentrations of L-theanine further intensified the effect on growth and spore production. In human and animal research, L-theanine can alleviate or eliminate inflammation in humans or animals, which means it has a significant inhibitory effect on microorganisms [25,26,27]. In plant diseases, L-theanine can inhibit the growth rate of *Colletotrichum coccodes* [28]. Therefore, combining the results of the present research, L-theanine not only inhibits fungal mycelium growth but also affects spore formation.

The functions of fungal genes include encoding proteins, regulating gene expression, and participating in cellular signaling [39]. Among them, the gene-encoding proteins are the most important part of the fungal genome. These genes produce functional proteins through transcription and translation processes to participate in the growth and development of fungi; β-1,3-glucan is crucial for the rigid structure of the cell wall in *Aspergillus fumigatus* and participates in the process of conidia germination and hyphal branching [40]. The SUN protein, from the glucan hydrolase family (GH132), plays a role in cell wall morphogenesis, binding and hydrolyzing β-(1,3)-glucan in a very specific way [33]. The genome of *A. fumigatus* contains two SUN genes: *SUN1* and *SUN2* [41,42]. Only *SUN1* plays a role in morphogenesis [41]. In the absence of SUN1, the hyphae of *A. fumigatus* swell, there is tip leakage and double-layer cell walls develop, indicating that SUN1 is necessary for normal growth and correct hyphal morphogenesis [41]. There are also studies indicating that the GH55, GH16, and GH81 families are necessary for the morphogenesis of conidia [40,43]. *SPH3* encodes a glycoside hydrolase, and the absence of *SPH3* can lead to a complete loss of detectable cell-wall-associated and -secreted GAG [44]. Chitin is crucial for forming the rigid structure of cell walls, maintaining the shape of fungal cells, and virulence [45]. Deficiency of multiple genes can lead to hindered chitin synthesis, morphological defects in hyphae and conidia, and weakened pathogenicity [46]. In the present study, low concentrations of L-theanine (Th2) primarily influenced molecular functions such as catalytic activity and oxidoreductase activity. High concentrations of L-theanine (Th4) not only affected molecular functions, but also affected certain biological processes. These processes include external encapsulating structure organization, cell wall organization or biogenesis, and cellular amino acid metabolic process, as revealed by the gene ontology analysis. Thus, these GO terms were suggested to play vital roles in the inhibition of L-theanine to *P. theae*.

Some representative genes such as aspartyl protease gene (TRINITY_DN8588_c0_g1), thioredoxin gene (TRINITY_DN10867_c0_g1), calreticulin gene (TRINITY_DN6207_c0_g1), and glyco_hydro_3 gene (TRINITY_DN13678_c0_g1) were affected by L-theanine. Thioredoxins are small enzymes that participate in redox reactions via reversible oxidation [47]. TRINITY_DN13678_c0_g1 belongs to the glycosyl hydrolase family and participates in relevant hydrolysis processes [48]. TRINITY_DN12261_c0_g1 was suppressed by L-theanine and was annotated as a heterokaryon incompatibility protein that was related to the viable heterokaryotic fungal cell. Based on KEGG analysis, carbohydrate-related metabolism, amino acid-related metabolism, and various types of N-glycan biosynthesis were significantly affected. The glyco_hydro-related gene involved in N-glycan biosynthesis and three annotated genes were affected. Aromatic amino acid aminotransferase and lyase gene (TRINITY_DN23344_c0_g1 and TRINITY_DN8664_c0_g1), amino acid transporters (TRINITY_DN6763_c0_g2), and three cytochrome P450 genes (TRINITY_DN16026_c0_g1, TRINITY_DN10916_c0_g1, and TRINITY_DN9904_c0_g2) were affected by L-theanine. Aromatic amino acid aminotransferases participate in many metabolic pathways such as methionine, phenylalanine, phenylalanine, and other forms of amino acid and alkaloid biosynthesis [49,50,51]. The cytochromes P450 play an important role in the biosynthesis and degradation of endogenous compounds and the biodegradation and detoxification of most exogenous compounds [52].

In summary, the present study illustrated that L-theanine had a significant effect on the pathogenic growth and formation of pathogenic spores, with distinct effects observed at different concentrations of L-theanine. The higher the concentration of L-theanine, the more obvious the effect on the growth and sporulation ability of the pathogen. Transcriptomic data indicated that the molecular pathways of low concentrations of L-theanine on the pathogen of tea grey blight disease are involved in pentose and glucuronate interconversions, isoquinoline alkaloid biosynthesis, starch and sucrose metabolism, metabolism of various amino acids such as histidine, tryptophan, phenylalanine, tyrosine, beta-alanine, etc. At high concentrations of L-theanine, some cellular structure-related metabolic activities were significantly affected, such as external encapsulating structure organization, cell-wall organization or biogenesis, and cellular amino acid metabolic processes. Thus, this study primarily demonstrated that L-theanine acts on the amino acid metabolism, carbohydrate metabolism, and cellular structure-related biosynthesis processes in pathogenic fungi. This work not only provides insights into the direct control effect of L-theanine on tea grey blight disease, but also reveals the potential molecular mechanisms of L-theanine on the pathogen of tea grey blight disease.

## 4. Materials and Methods

### 4.1. Fungal Materials and Treatments

The pathogenic fungus of tea grey blight, *P. theae,* was isolated, purified, and analyzed by internal transcribed spacer (ITS) sequencing. After the pathogen was first cultured in potato dextrose agar (PDA) medium under 28 °C for 3 days, the fungal cakes (about 0.5 cm in size) were prepared and inoculated into a PDA medium containing different concentrations of L-theanine, below 28 °C. L-theanine concentrations were set as 0 (Th0), 20 mg/mL (Th2), and 40 mg/mL (Th4). A Vernier caliper was used to continuously measure the colony diameter of pathogenic fungus cultured in PDA plates with different concentrations of L-theanine. At the same time, fungal samples were obtained by scraping the mycelium from the culture plates for transcriptome analysis on day 5, below 28 °C. The fungus was continuously cultured for spore collection and observation for 12 days. The spore count was calculated using a blood cell counting plate (16 × 25). Three biological replicates were collected per sample. The pathogenic mycelium samples were frozen in liquid nitrogen, roughly ground, and kept at −80 °C for further research.

### 4.2. RNA Extraction, Library Preparation, and Sequencing

The isolation and purification of pathogenic fungal mycelium’s RNA, construction of cDNA libraries, and sequencing were performed (Sangon Biotech (Shanghai) Co., Ltd., Shanghai, China). The total RNA of mycelium was extracted using the Total RNA Extractor (Trizol) kit (B511311, Sangon, Shanghai, China), according to the manufacturer’s protocol, and genomic DNA contamination was removed by RNase-free DNase I. The quality and quantity evaluation of RNA, library preparation, and sequencing of all RNA samples were performed by the Sangon Biotech (Shanghai) Co., Ltd. The detailed sequencing program and platform information referred to the previously published manuscript [53].

### 4.3. Data Assessment and Quality Control

The quality of sequenced data was evaluated through Fast QC (version 0.11.2). Raw reads were filtered by Trimmomatic (version 0.36) by removing the adaptor sequence, the low-quality bases (Q < 20), the base value less than 20 of reads tail (window size = 5 bp), and the reads and pairing reads with reads length less than 35 nt. The remaining clean data were used for further analysis.

### 4.4. Assemble, Annotate, and Map Transcript

The clean data were assembled into transcripts using Trinity with parameters min_kmer_cov 2, and an elimination of redundancy of the transcripts was carried out. The longest transcript in each transcript cluster was taken as unigene. The unigene was used as a reference sequence for subsequent analysis. NCBI Blast+ was used to compare transcripts with CDD, KOG, COG, NR, NT, PFAM, and other databases to obtain functional annotation information. KEGG (Kyoto Encyclopedia of Genes and Genomes) annotation information of the transcript was obtained by using KAAS (KEGG Automatic Annotation Server). CDS prediction was made based on comparison results with the transcripts database and TransDecoder. Bowtie2 was used to compare the valid data of all samples to the transcripts obtained by splicing, and statistical mapping information was obtained. RSeQC was used to analyze the redundant sequence and insert fragment distribution according to the comparison results. BEDTools was used to check the uniformity of distribution and statistical analysis of gene coverage. SSR analysis was performed using MISA based on the transcript sequence information obtained by splicing.

### 4.5. Expression Analysis

Gene expression values of the transcripts were computed by StringTie (version 1.3.3b). Based on the representation matrix of samples, we conducted multi-direction statistical analysis and exploration such as sample comparison analysis. Principal component analysis (PCA) and principal co-ordinates analysis (PCoA) were performed to reflect the distance and difference between all samples. The TPM (transcripts per million) eliminated the influence of gene lengths and sequencing discrepancies to enable direct comparison of gene expression between samples. DESeq2 (version 1.12.4) was used to determine differentially expressed genes (DEGs) between two samples. Genes were considered as significantly differentially expressed if *q*-value ≤ 0.001 and |FoldChange| ≥ 2. Gene expression differences were visualized by scatter plot and volcano plot.

### 4.6. Functional Analysis of Differentially Expressed Genes

Functional enrichment analyses including GO and KEGG were performed to identify which DEGs were significantly enriched in GO terms or metabolic pathways. GO terms and KEGG pathways with a false discovery rate (*q*-value) < 0.05 were considered as significantly altered. The correlation analysis network map was drawn based on the results of gene function enrichment analysis.

### 4.7. Quantitative Real-Time PCR (qRT-PCR) Validation

Total RNA extraction was as described previously. PrimeScript II First Strand cDNA Synthesis Kit (Takara, Ishiyama, Japan) was used for reverse transcription. Eight genes of the pathogen were selected for qRT-PCR with specific primers (Table S10). The qRT-PCR was performed with a Roche LightCycler 96 system (Roche Applied Science, Mannheim, Germany) with TB Green SYBR Kit (Takara, Ishiyama, Japan). The amplification system and program were performed according to the manufacturer’s protocol. The 2^−ΔΔct^ method was performed to analyze relative quantitative data with a reference gene, β-actin. Three experimental replicates and technical replicates of each sample were performed to ensure reproducibility and reliability.

### 4.8. Statistical Analysis

SPSS 22.0 was used for statistical analysis. The levels of statistical significance were determined according to least significant difference (*p*-value < 0.05).

## Data Availability

The data that support the findings of this study are available from the corresponding author (F.C.), upon reasonable request.

## Supplementary Materials

The following supporting information can be downloaded at: https://www.mdpi.com/article/10.3390/ijms25063493/s1.
