# Supplementary material for: The Response of Growth and Transcriptome Profiles of Tea Grey Blight Disease Pathogen Pestalotiopsis theae to the Variation of Exogenous L-Theanine
Source: Int J Mol Sci. 2024 Mar 20;25(6):3493. doi: 10.3390/ijms25063493 (PMC10971465; doi:10.3390/ijms25063493)
Supplement: Supplementary file 1 [file ijms-25-03493-s001.zip › ijms-2863686-supplementary/Supplementary Figures S1-S15.pdf]

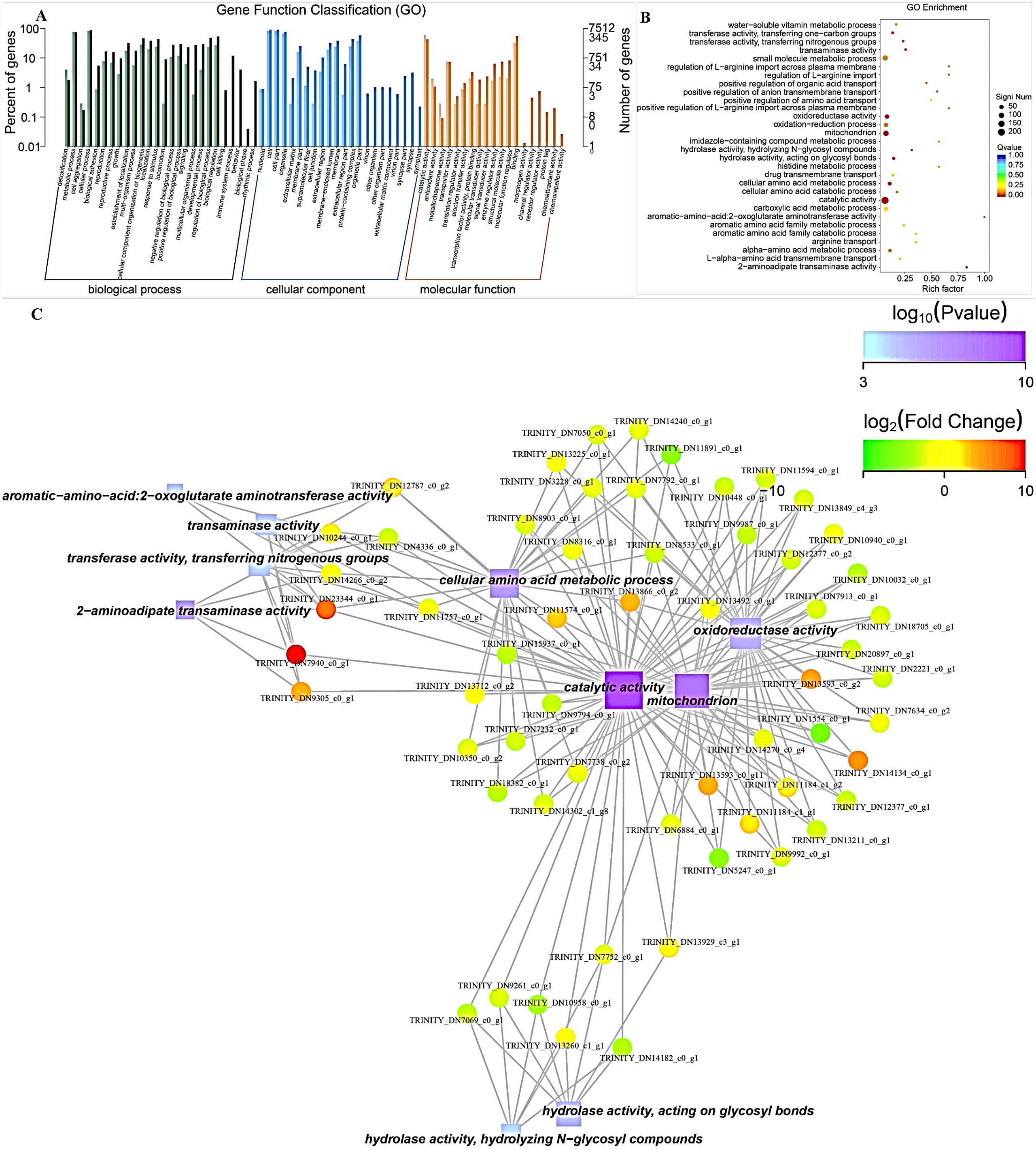

Figure S1. GO analysis of DEGs in Th2 *vs.* Th0 comparison

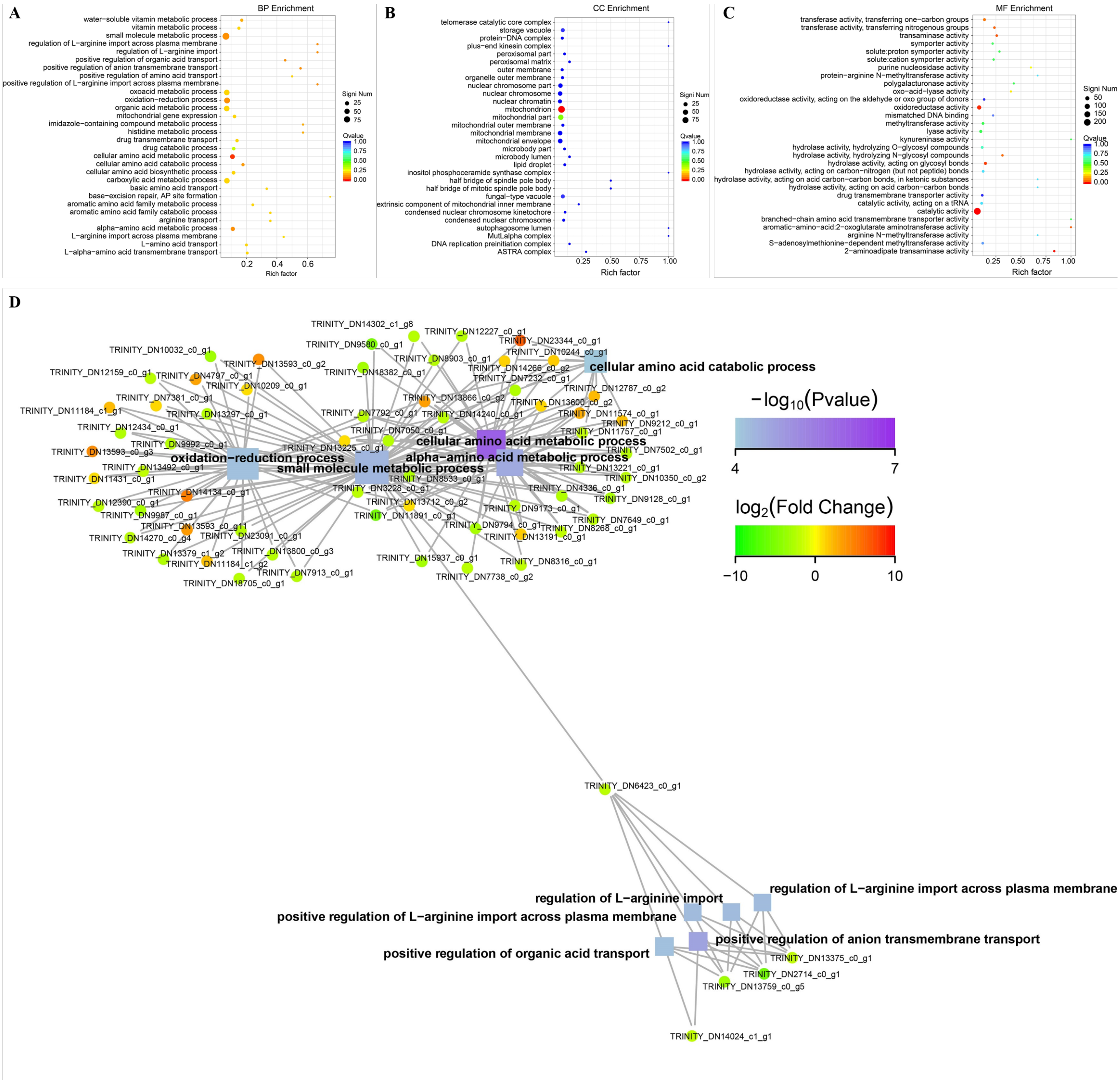

Figure S2. BP/CC/MF analysis and gene correlation network of DEGs in Th2 *vs.* Th0 comparison

A

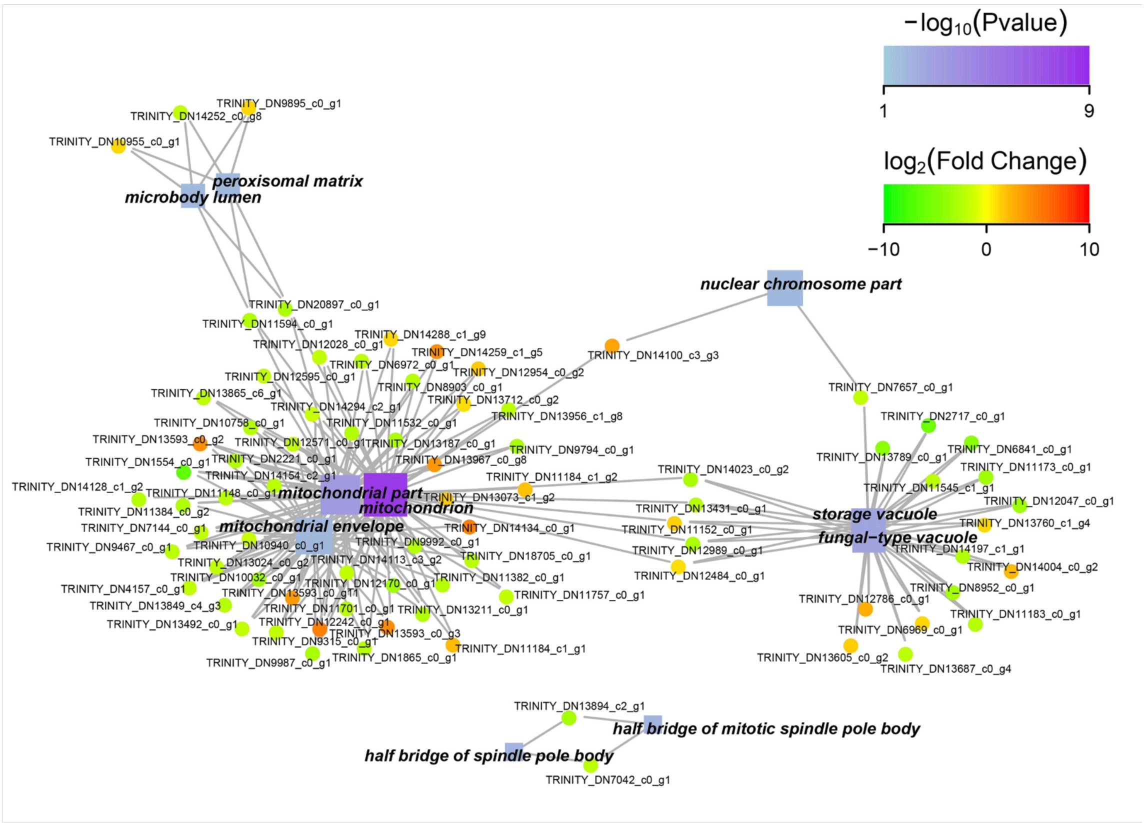

B

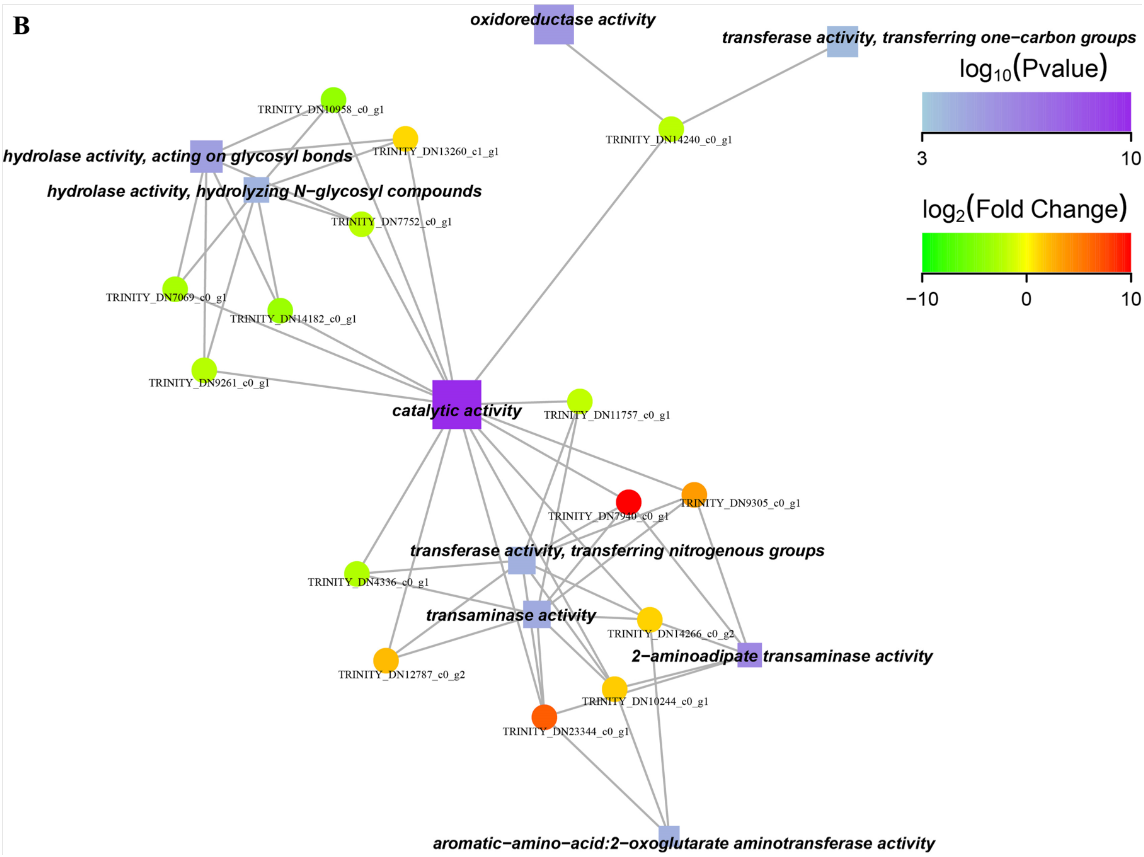

Figure S3. Correlation network of DEGs involved in CC and MF in Th2 *vs.* Th0 comparison

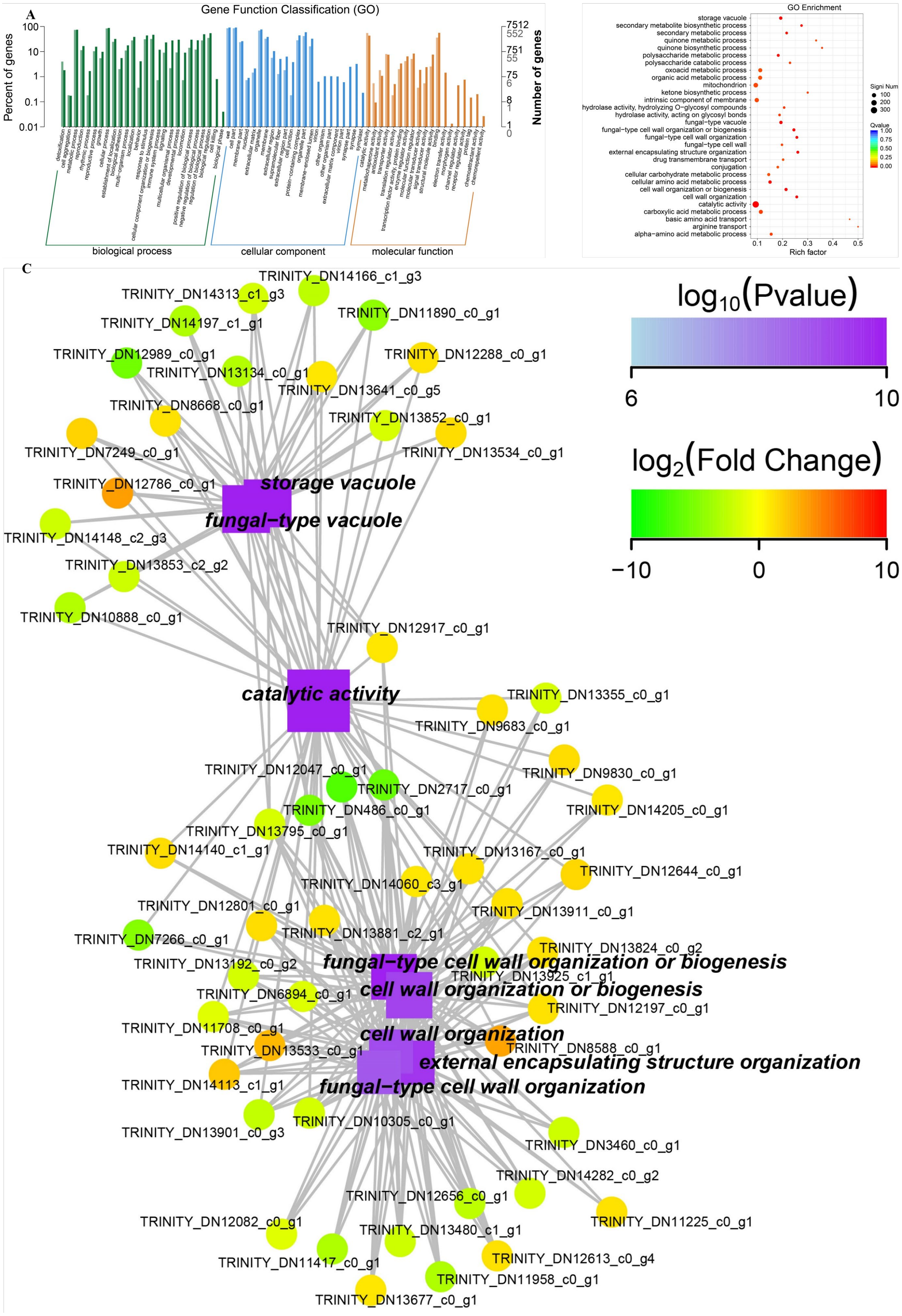

Figure S4. GO analysis of DEGs in Th4 *vs.* Th0 comparison

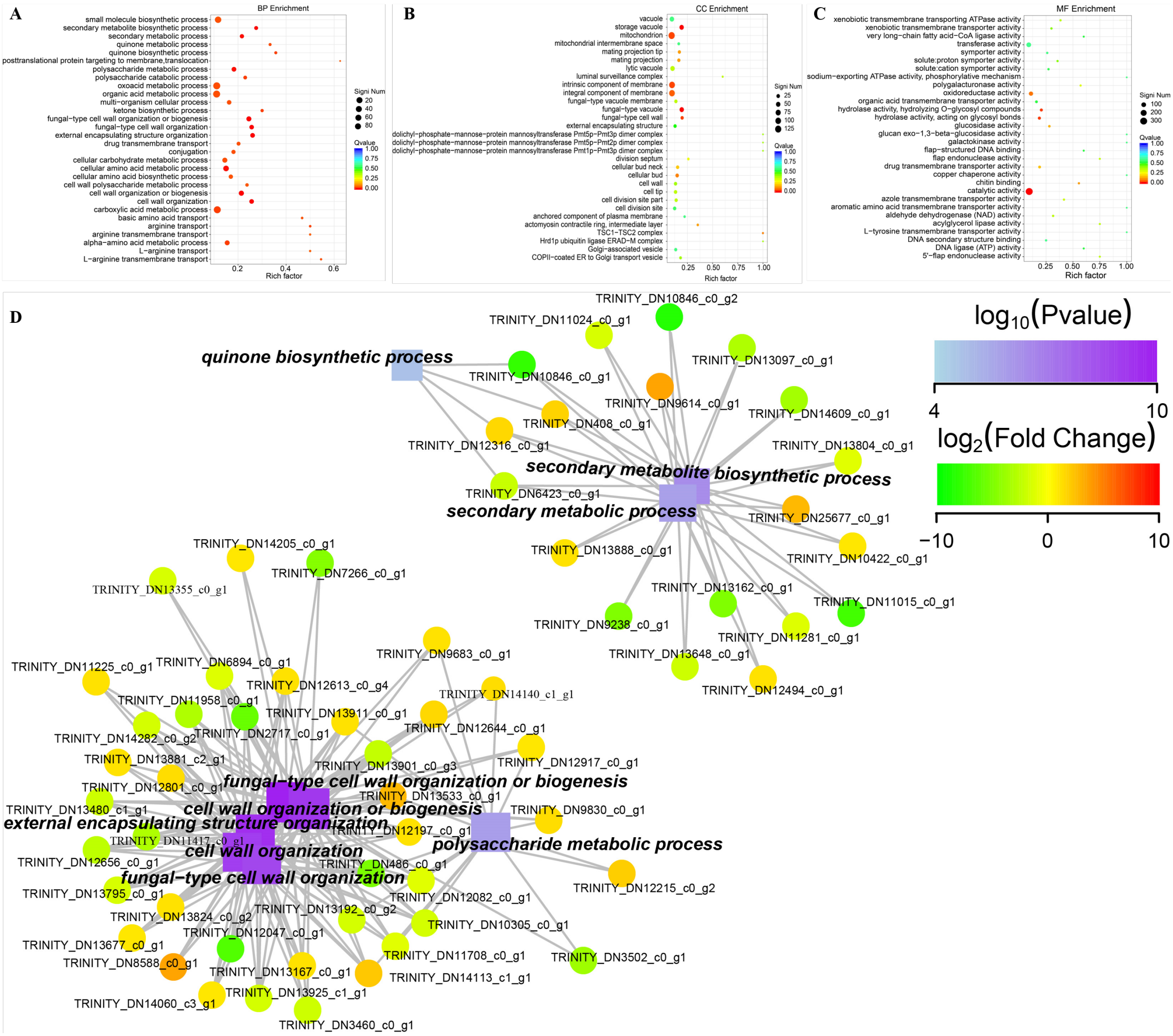

Figure S5. BP/CC/MF analysis and gene correlation network of DEGs in Th4_vs_Th0 comparison

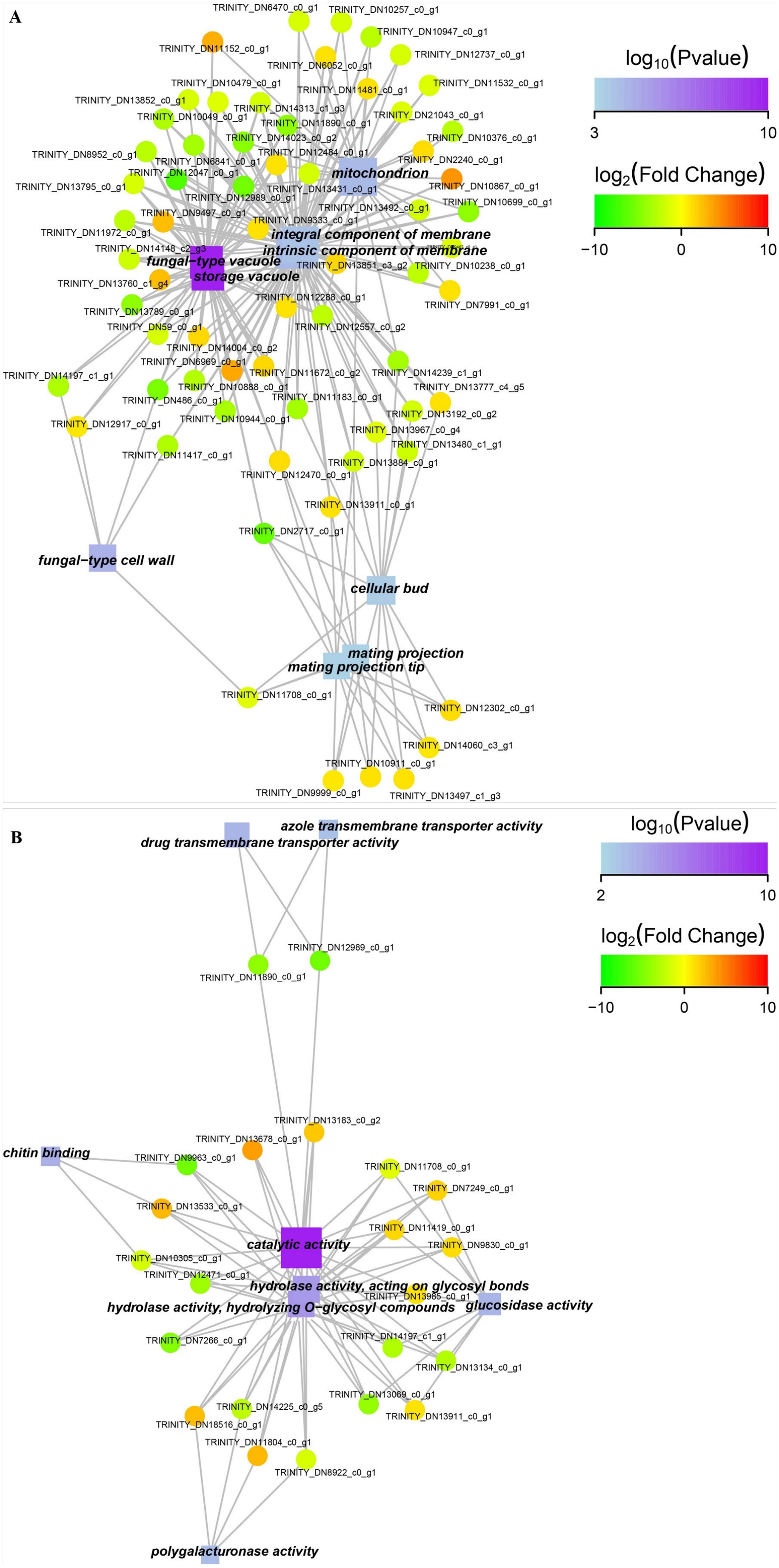

Figure S6. Correlation network of DEGs involved in CC and MF in Th4 *vs.* Th0 comparison

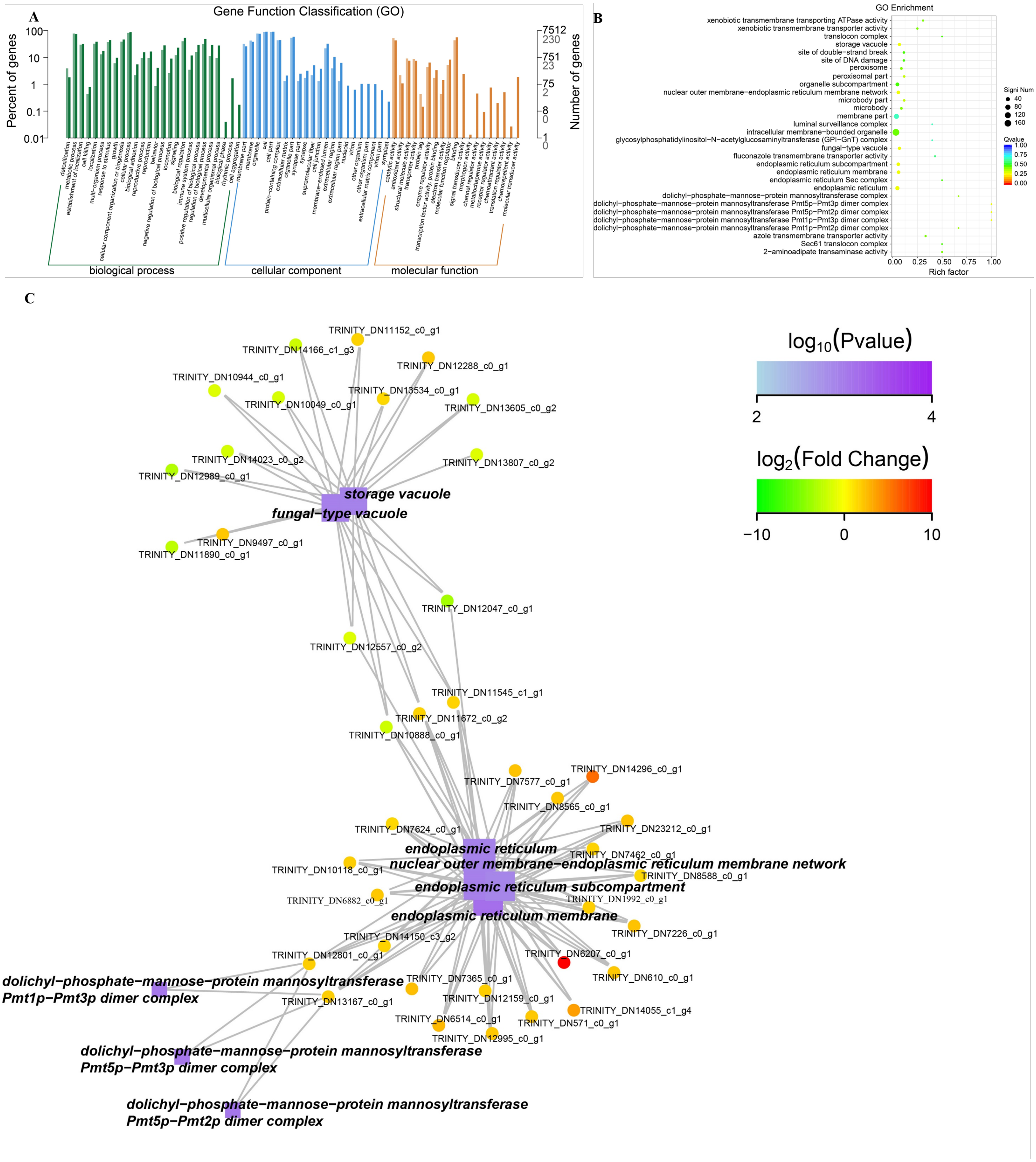

Figure S7. GO analysis of DEGs in Th4 *vs.* Th2 comparison

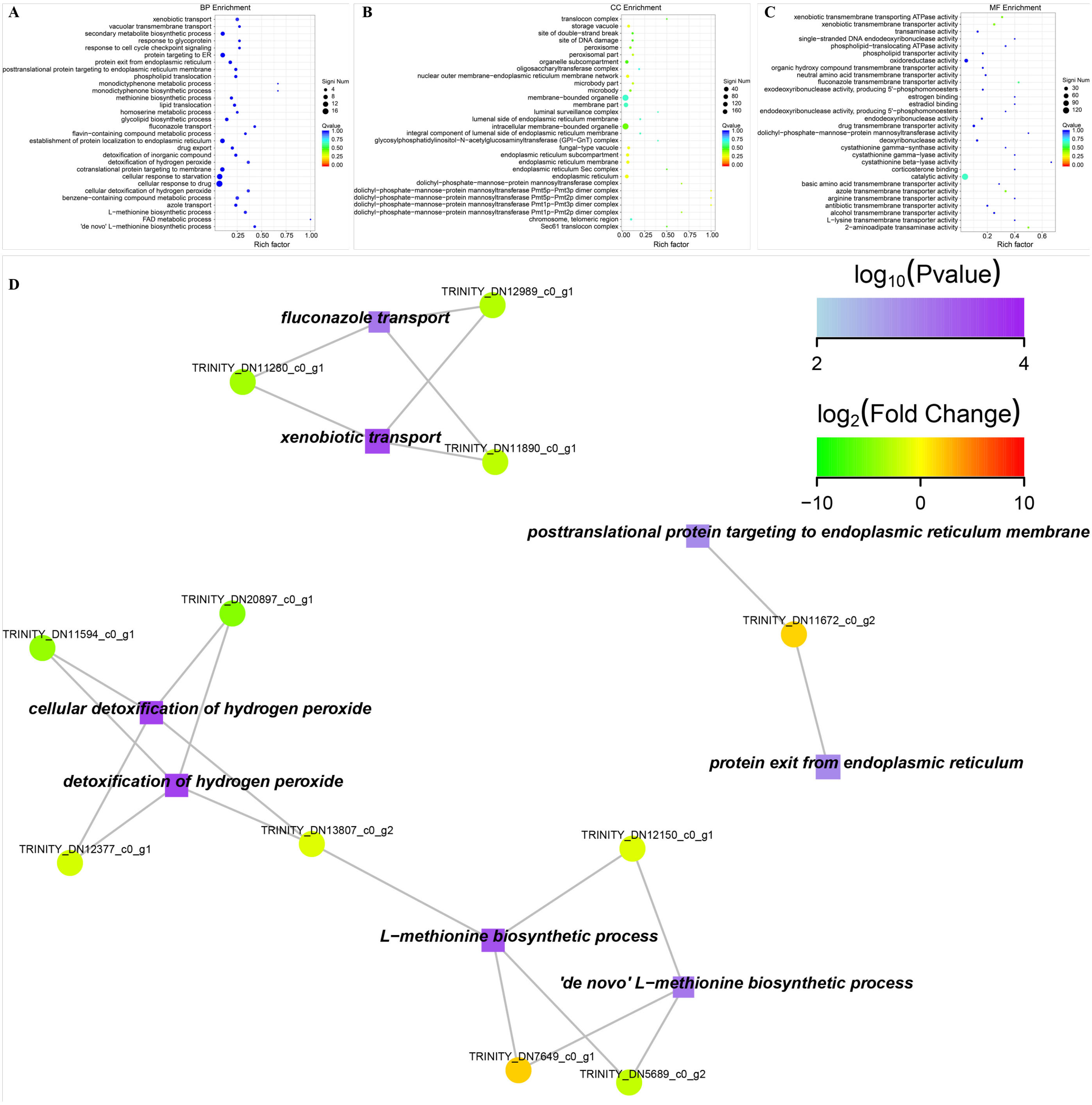

Figure S8. BP/CC/MF analysis and gene correlation network of DEGs in Th4 *vs.* Th2 comparison

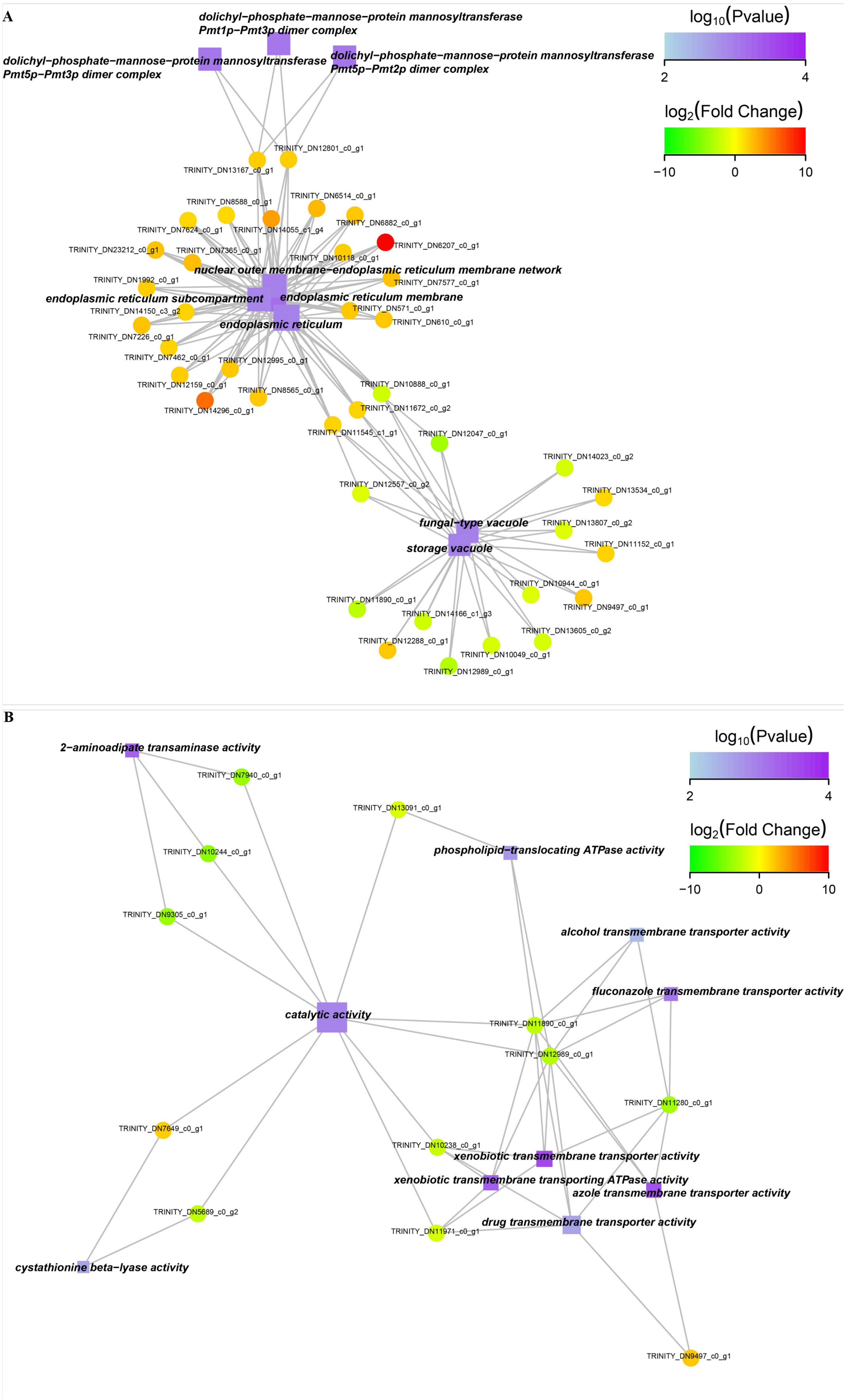

Figure S9. Correlation network of DEGs involved in CC and MF in Th4 *vs.* Th2 comparison

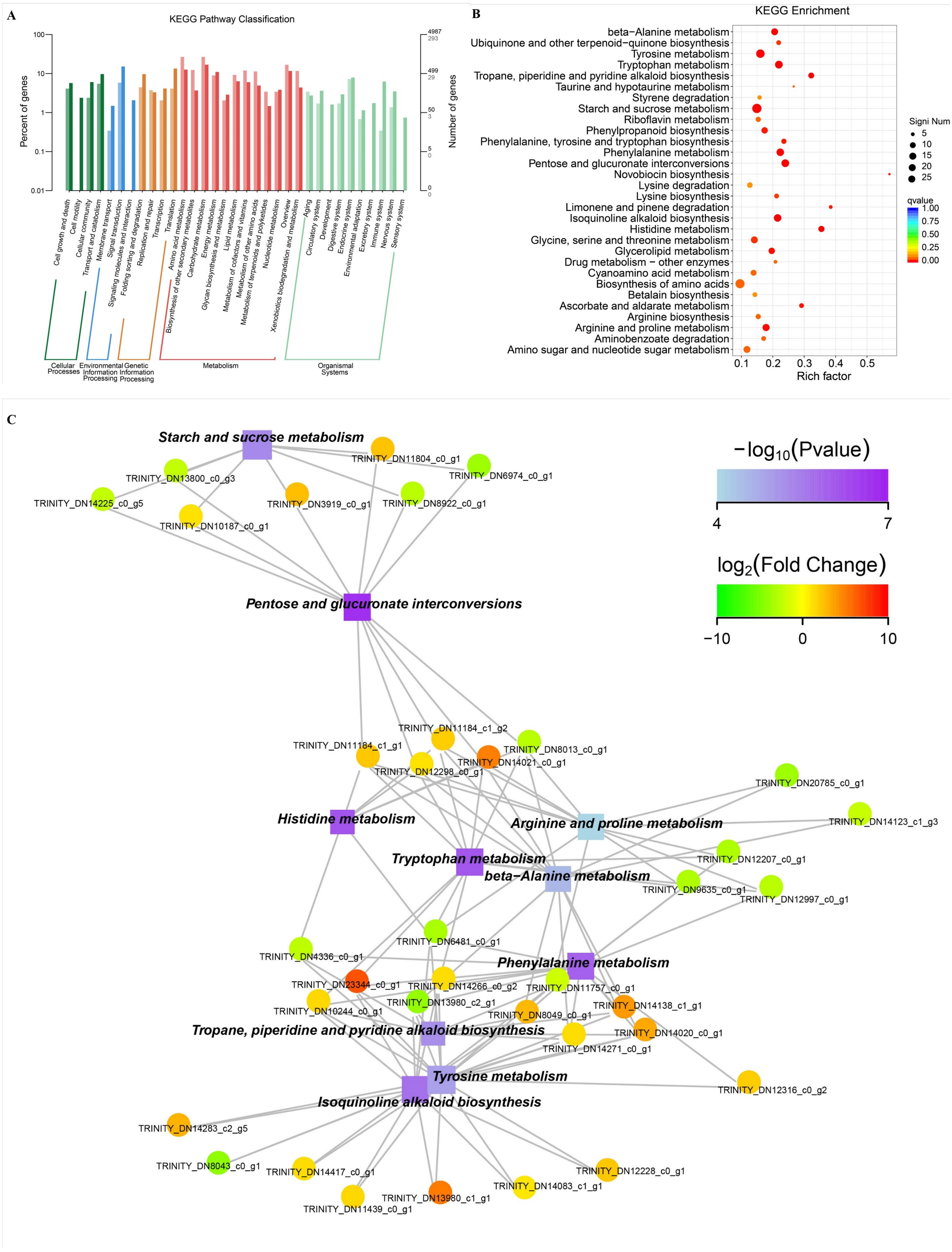

Figure S10. KEGG analysis of DEGs in Th2 *vs.* Th0 comparison

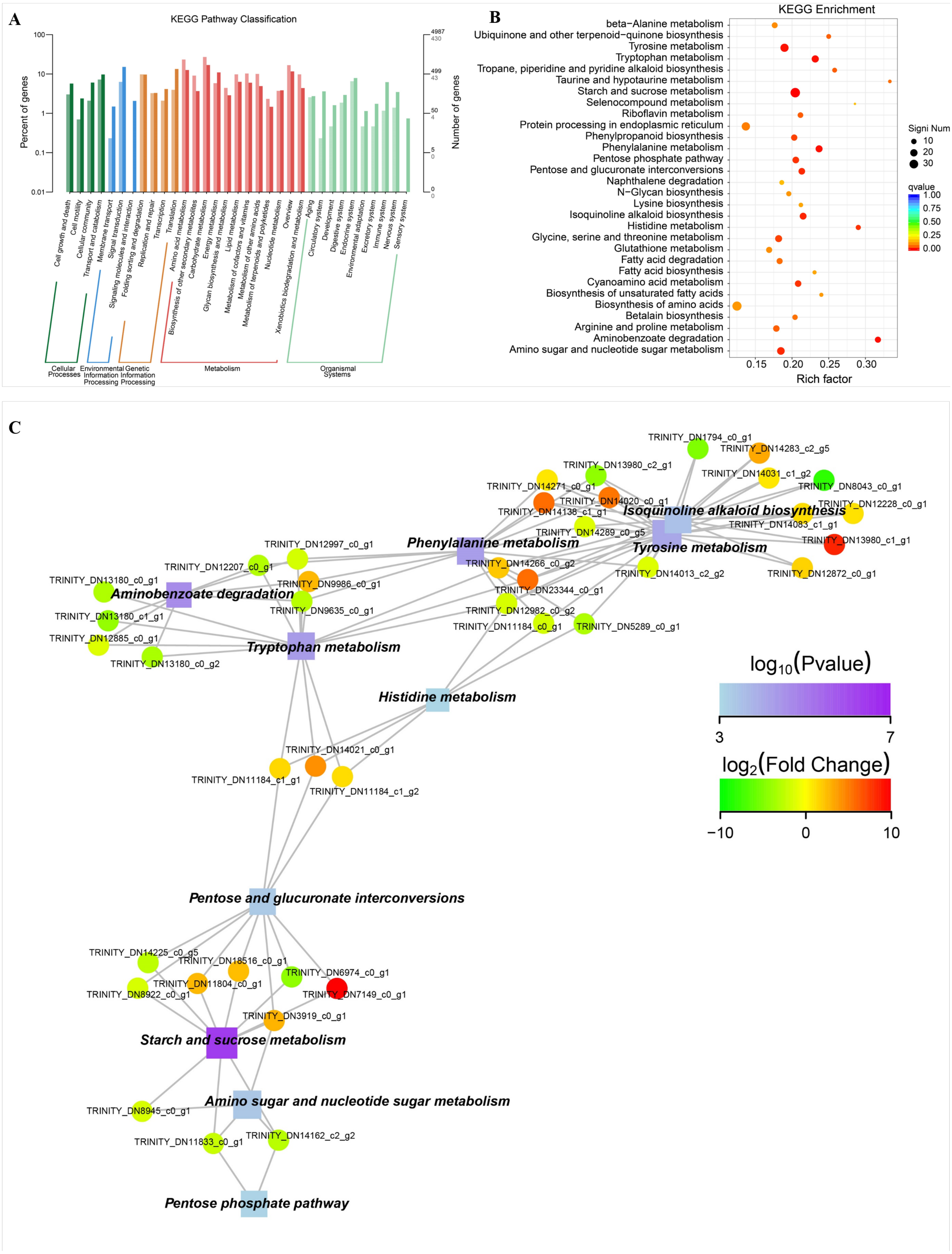

Figure S11. KEGG analysis of DEGs in Th4 *vs.* Th0 comparison

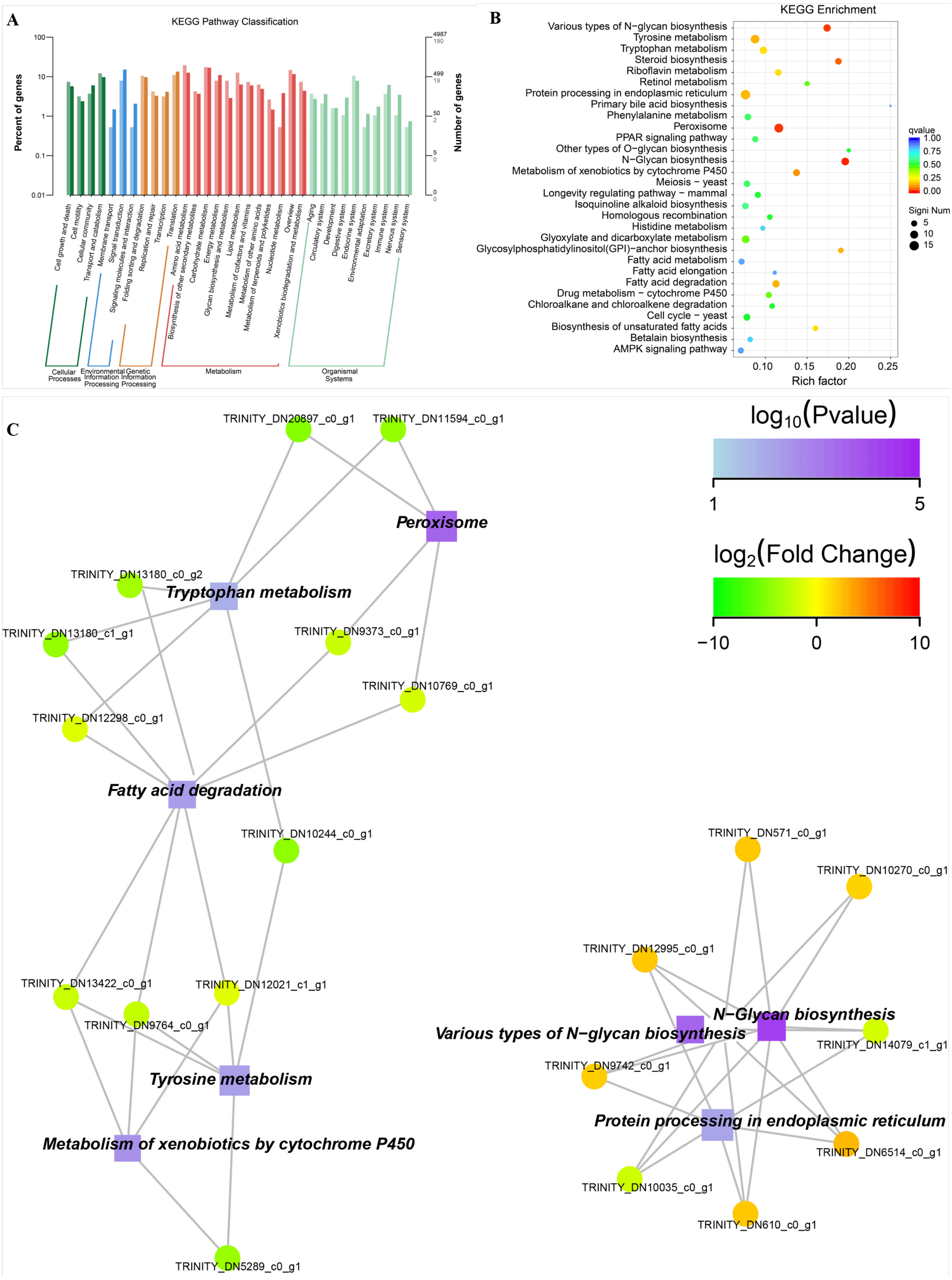

Figure S12. KEGG analysis of DEGs in Th4 *vs.* Th2 comparison

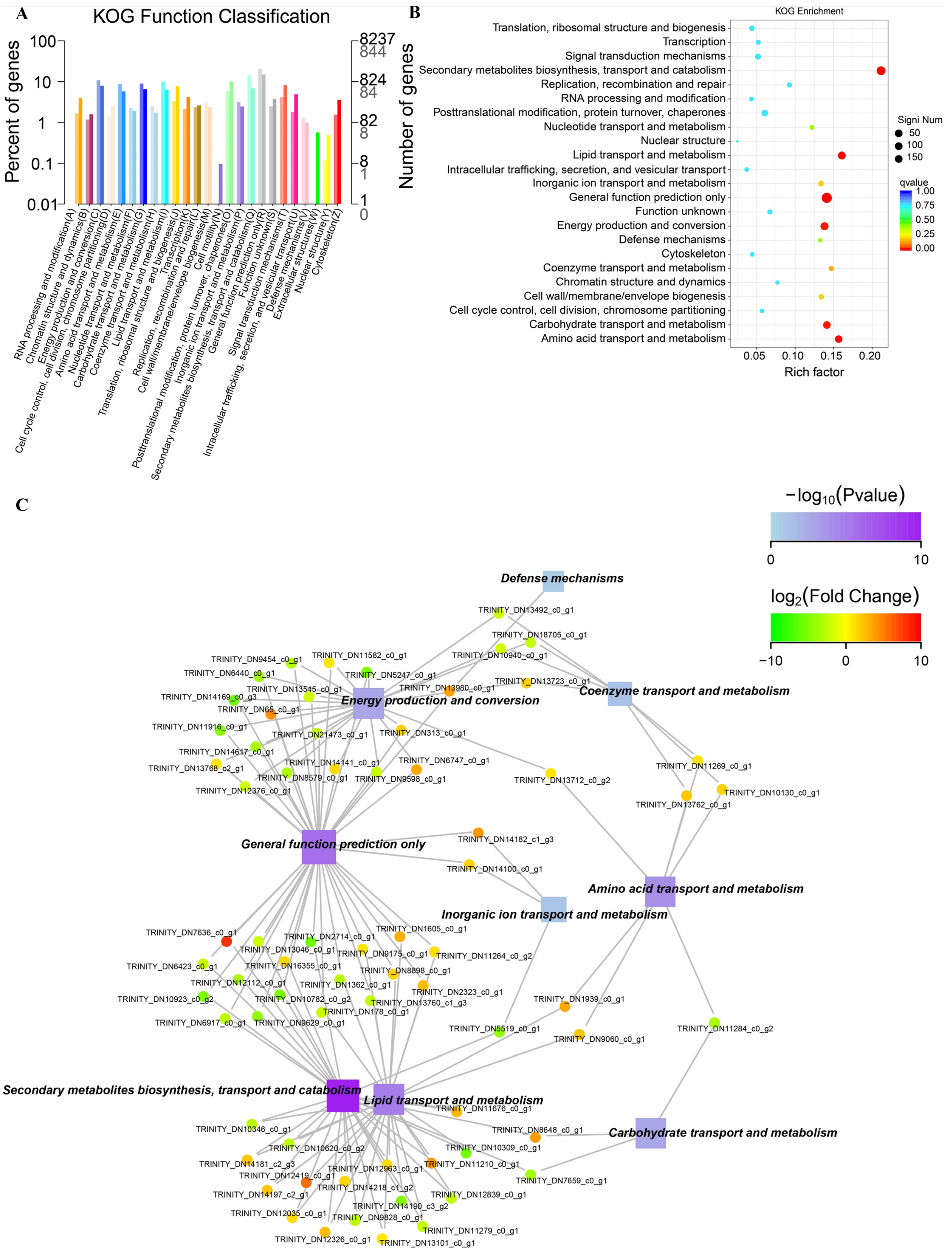

Figure S13. KOG analysis of DEGs in Th2 *vs.* Th0 comparison

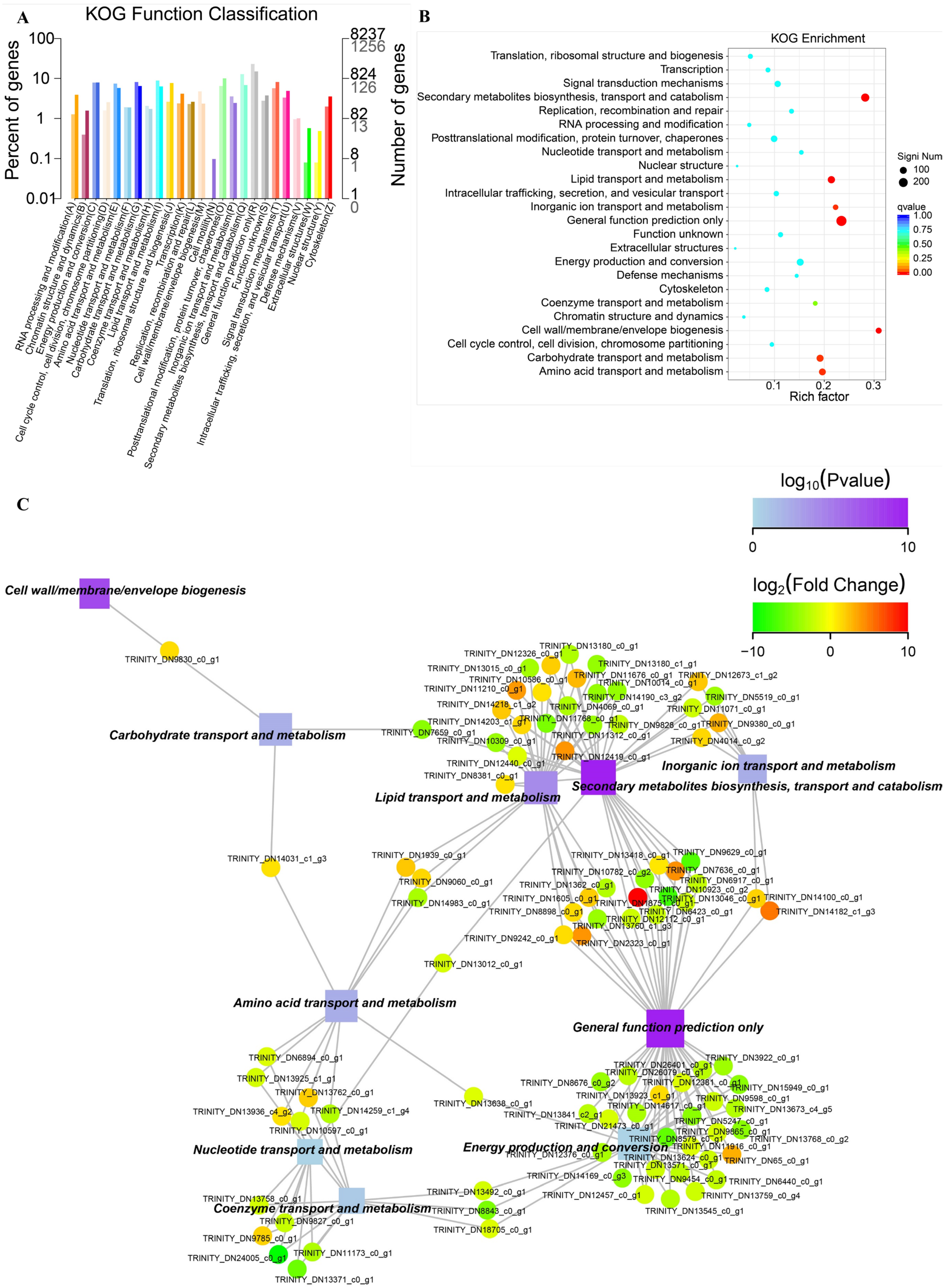

Figure S14. KOG analysis of DEGs in Th4 *vs.* Th0 comparison

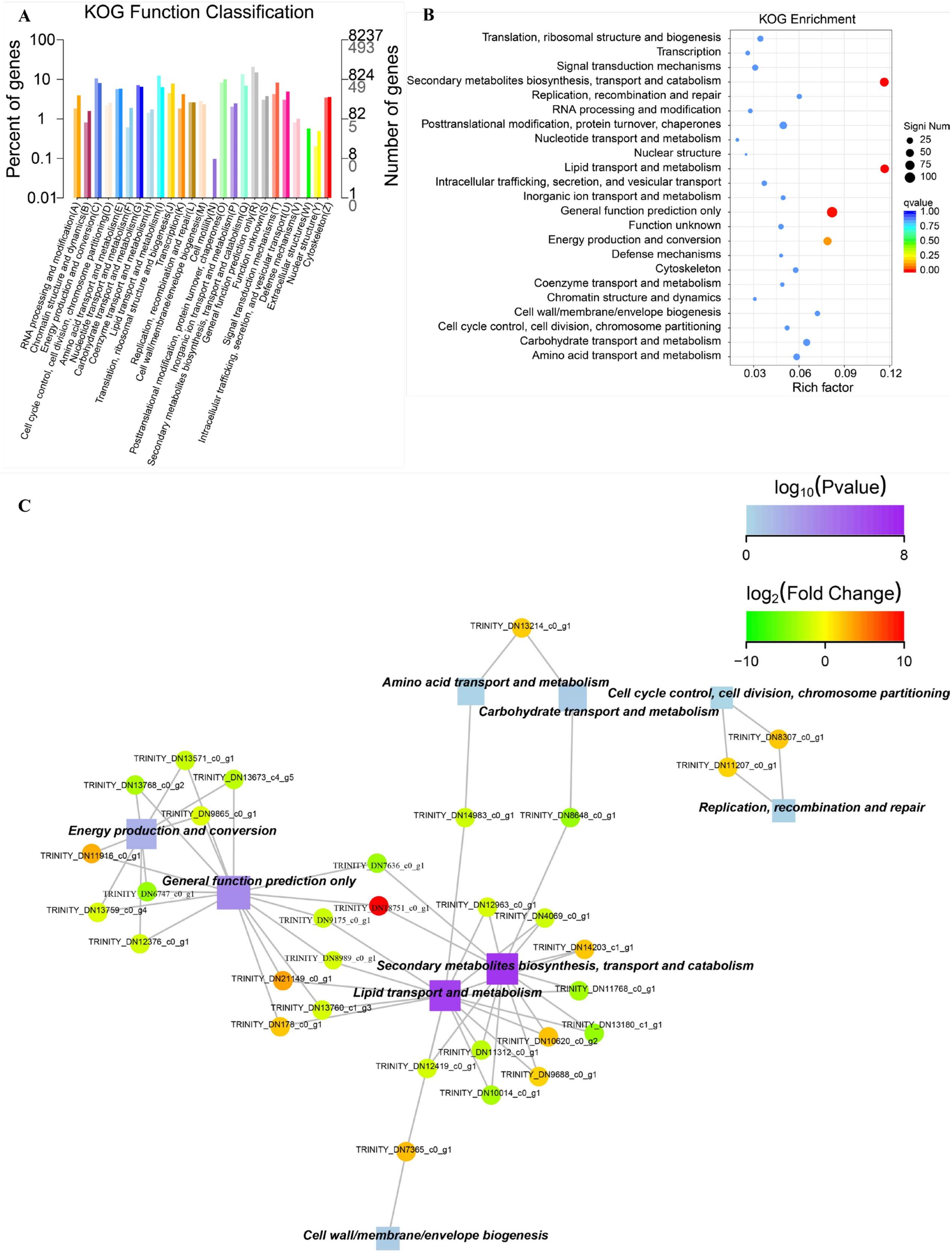

Figure S15. KOG analysis of DEGs in Th4 *vs.* Th2 comparison
